# Light Supplementation in Pitaya Orchards Induces Pitaya Flowering in Winter by Promoting Phytohormone Biosynthesis

**DOI:** 10.3390/ijms25094794

**Published:** 2024-04-27

**Authors:** Meng Wang, Jiaxue Li, Tao Li, Shaoling Kang, Senrong Jiang, Jiaquan Huang, Hua Tang

**Affiliations:** 1 Sanya Institute of Breeding and Multiplication, Hainan University, Sanya 572025, China; 21110901000038@hainanu.edu.cn (M.W.); jiaxue2046@outlook.com (J.L.); ltsz0959@eav.top (T.L.); 21210901000021@hainanu.edu.cn (S.K.); 21220951310031@hainanu.edu.cn (S.J.); 2 School of Tropical Agriculture and Forestry, Hainan University, Haikou 570228, China

**Keywords:** phytohormones, pitaya, flowering, supplementary lighting, induced flowering

## Abstract

The interaction between light and phytohormones is crucial for plant growth and development. The practice of supplementing light at night during winter to promote pitaya flowering and thereby enhance yield has been shown to be crucial and widely used. However, it remains unclear how supplemental winter light regulates phytohormone levels to promote flowering in pitaya. In this study, through analyzing the transcriptome data of pitaya at four different stages (NL, L0, L1, L2), we observed that differentially expressed genes (DEGs) were mainly enriched in the phytohormone biosynthesis pathway. We further analyzed the data and found that cytokinin (CK) content first increased at the L0 stage and then decreased at the L1 and L2 stages after supplemental light treatment compared to the control (NL). Gibberellin (GA), auxin (IAA), salicylic acid (SA), and jasmonic acid (JA) content increased during the formation of flower buds (L1, L2 stages). In addition, the levels of GA, ethylene (ETH), IAA, and abscisic acid (ABA) increased in flower buds after one week of development (L2f). Our results suggest that winter nighttime supplemental light can interact with endogenous hormone signaling in pitaya, particularly CK, to regulate flower bud formation. These results contribute to a better understanding of the mechanism of phytohormone interactions during the induction of flowering in pitaya under supplemental light in winter.

## 1. Introduction

In angiosperms, the transition of plants from the stage of vegetative growth to reproductive development is the vital aspect of their life cycle [1]. The process of flower formation in plants is divided into three stages: floral induction, floral evocation, and floral development. Flowering is a complex physiological process influenced by various signals, including exogenous factors like photoperiod and temperature, as well as endogenous signals like phytohormones [2,3,4,5]. Currently, researchers have identified six signaling pathways in *Arabidopsis* that regulate flowering in plants. These include the photoperiodic, vernal, autonomous, gibberellin, temperature-sensitive, and age regulatory mechanisms [5,6,7,8]. These pathways interact with each other and collectively regulate flowering time in *Arabidopsis*. These include FLOWERING LOCUS T (*FT*), SUPPRESSOR OF OVEREXPRESSION OF CONSTANS 1 (*SOC1*), and LEAFY (*LFY*), which combine signals from different flowering pathways and send them to floral meristem genes including FRUITFULL (*FUL*), APETALA1 (*AP1*), and *LFY* [1,8,9,10]. The precise management of *Arabidopsis* blooming time is achieved by activating these genes to regulate the development of floral primordia on the lateral side of inflorescence meristem tissue.

The function of plant hormones has been clarified through studies involving the application of exogenous hormones and the analysis of relative biosynthetic signaling mutants [11,12]. Organic signaling molecules generated by a plant’s metabolism are called endogenous plant hormones. Even at very low doses, they can have a considerable physiological impact. These hormones form a complex and comprehensive signaling network in the plant, regulating plant growth and development both independently and synergistically [13]. At the moment, the most intensively studied plant hormones include cytokinin (CK), brassinosteroid (BR), auxins (IAA), strigolactone (SL), gibberellin (GA), ethylene (ETH), jasmonic acid (JA), salicylic acid (SA), and abscisic acid (ABA).

The GA pathway is one of the first six floral pathways identified. For instance, DELLA directly binds to SQUAMOSA PROMOTER BINDING-LIKE (*SPL*) transcription factors that are targeted by microRNA156 (miR156) in *Arabidopsis*. These transcription factors activate the *MADS* box and *miR172* genes to induce flowering [14]. In addition, DELLA causes delayed flowering by repressing the activation of CONSTANS (*CO*), *WRKY12*, *WRKY13*, and *WRKY75* genes [15,16,17]. Zeatin is the primary natural active component of CK, and it exists in two isomeric forms: trans-zeatin (tZ) and cis-zeatin (cZ). CK accelerates DNA replication in pre-cell division and lays the groundwork for subsequent cell division [18]. It has been demonstrated that the absence of GA or JA can result in short stamens and male sterility [13]. GA can induce the expression of *MYB21*/*24*/*57* by promoting the synthesis of JA, thereby promoting stamen development [19]. Additionally, JA can inhibit the transcription of FLOWERING LOCUS T (*FT*) by activating the transcription factors *MYC2*, *MYC3*, and *MYC4*, thereby delaying the flowering time in plants [20]. ETH plays a crucial role in determining gender. For instance, *CsWIP1* inhibits male flower development in cucumber by suppressing ethylene synthesis, while ETH promotes female flower development by inducing apoptosis in stamens [21]. Recent studies have found that ETH produced in melon carpels activates *CmHB40* expression through the *CmEIN3*/*CmEIL1* module in the stamen primordia, leading to stamen arrest [22]. SL is also involved in plant flowering and has been shown to inhibit flowering by suppressing melatonin synthesis [23]. In addition, growth hormone, ABA, and SA often synergize with other hormones to regulate plant flowering. For example, JA not only regulates the biosynthesis of IAA by activating the expression of ASA1, but also influences the polar transport of growth hormone [24].

Pitaya is a climbing fruit tree belonging to the genus *Hylocereus* or *Selenicereus* in the Cactaceae family [25]. Pitaya is a major crop in Brazil, Ecuador, Colombia, Costa Rica, and Mexico. It is endemic to tropical and subtropical rainforests, mostly in Central America [26]. It is extensively grown in both tropical and subtropical areas, particularly in China, Vietnam, Malaysia, Thailand, and the Philippines. Currently, China has over 66,700 hectares of pitaya planting area, making it the world’s largest pitaya planting area and providing significant economic returns to growers.

Pitaya is a long-day plant and will not flower if there are fewer than 12 h of sunlight [27]. During the growing season from April to October, pitaya flowers for about 30 to 35 days. The optimal temperature range for pitaya growth is between 25 °C and 35 °C. Once the temperature drops below 10 °C or rises above 38 °C, pitaya growth stops. In regions other than Hainan in China, the winter months from November to March are not only cold but also have short sunshine hours. The Hainan region has a tropical monsoon climate that meets the temperature requirements of pitaya. Additionally, the need for light can be fulfilled by providing supplemental light during nighttime. During this time, the flowering period of pitaya is usually 40 to 45 days. This allows for year-round fruit production and meets the Chinese market’s demand for pitaya during the winter months. However, supplementing with artificial light has the disadvantages of high cost and wastage of power resources. It has been demonstrated that plant flowering is synergistically regulated by both light and phytohormones [28]. Nevertheless, our understanding of the precise mechanisms by which phytohormones and light interact to control plant development and flowering remains incomplete. Therefore, in this study, we utilized RNA-seq in conjunction with metabolomic analysis to examine the expression levels of phytohormone-related genes and phytohormones, the synergistic effects among phytohormones, and phytohormone signaling pathways during light supplementation-induced floral formation. Next, we will attempt to use exogenous phytohormones to shorten the duration of supplemental light in winter. This will provide theoretical guidance for the use of phytohormones in promoting pitaya winter flowering, enhancing floral regulation, and improving future production.

## 2. Results

### 2.1. Light and Hormone-Mediated Flowering in Pitaya

**Figure 1:**
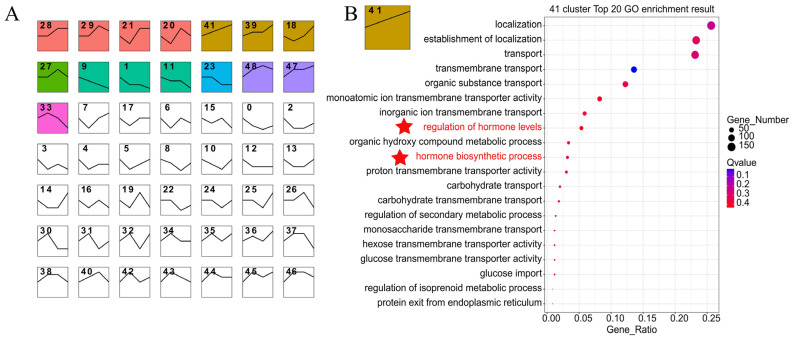
Dynamic Expression Pattern Analysis of DEGs in the Four Stages of Pitaya. (**A**): The trend depicted in the line graph represents the overall direction of gene expression over time in this cluster. Statistically significant clusters (*p* < 0.05) are highlighted with colored backgrounds, classifying them into 20 clusters. The identical background color indicates a similar trend, which is further categorized into 7 clusters. (**B**): GO enrichment analysis and functional annotation of 41 clusters. In the visual representation, red circles indicate up-regulated genes, with circle size corresponding to the number of genes, and pentagrams indicating genes enriched for key functions.

We analyzed the DEGs in the pitaya transcriptome at four stages by constructing co-expression profiles of NL, L0, L1, L2. The results showed that the DEGs at the four stages were classified into 20 different clusters (Figure 1A). We performed Gene Ontology (GO) functional annotation and enrichment analysis on the DEGs for each of the 20 different modes of variation (Figure 1 and Figure S1). The results revealed that phytohormone-related signaling pathways were enriched in clusters 41, 39, and 18 (Figure 1B and Figure S1E,F). These pathways include the regulation of phytohormone synthesis, phytohormone metabolism, response to stress, response to biotic, cellular response, and cellular response to chemical stimuli. The DEGs exhibited up-regulated expression patterns at all four stages. This result suggests that phytohormones not only mediate the plant stress response but may also play a crucial role in the stages of flower formation and floral organ development.

Furthermore, we conducted GO enrichment analysis on other clusters exhibiting a similar trend. The GO results for biological functions primarily focused on cellular component biogenesis, cell cycle, DNA replication, and RNA metabolism in clusters 28, 29, 21, and 20 (Figure S1A–D). This indicates that pitaya accelerated DNA and RNA synthesis and promoted cell division in pitaya after additional light supplementation. In addition, a similar upward trend was observed in 27 clusters (Figure S1G), and the GO results for biological functions were mainly concentrated in shoot system development, cell cycle processes, reproductive shoot system development, flower development, and sexual reproductive-related genes. This suggests that the plant’s cell cycle is accelerated after supplemental light, and the reproductive system of pitaya is strengthened, thereby favoring the transition from the nutrient stage to the reproductive stage. In clusters 48 and 47, the GO results mainly focused on nucleic acid metabolic processes, growth, and general metabolic processes (Figure S1L–M). This indicates that the metabolism related to the regulation of growth gradually slowed in plants after supplemental light, and mainly focused on the reproductive stage.

Similarly, we conducted GO enrichment for clusters exhibiting down-regulation trends. We found that clusters 9, 11, and 1 showed down-regulation in response to abiotic stimulus, response to light, post-embryonic development, reproductive development, and flower development (Figure S1H–J). These changes may be related to adaptive strategies in pitaya, such as reducing the response to abiotic stimuli under supplemental light conditions or adjusting the timing of reproductive development and flower opening. In the cluster of 23, L0–L1 was down-regulated (Figure S1K). The GO results indicated that the gene expression related to the obsolete oxidation reduction process and photosynthesis was also down-regulated. This suggests that after supplemental light, the plant can utilize the energy produced by photosynthesis to maintain normal metabolism and growth while simultaneously reducing the redox reaction to replenish energy.

### 2.2. Regulation of DEGs Related to Phytohormones

**Figure 2:**
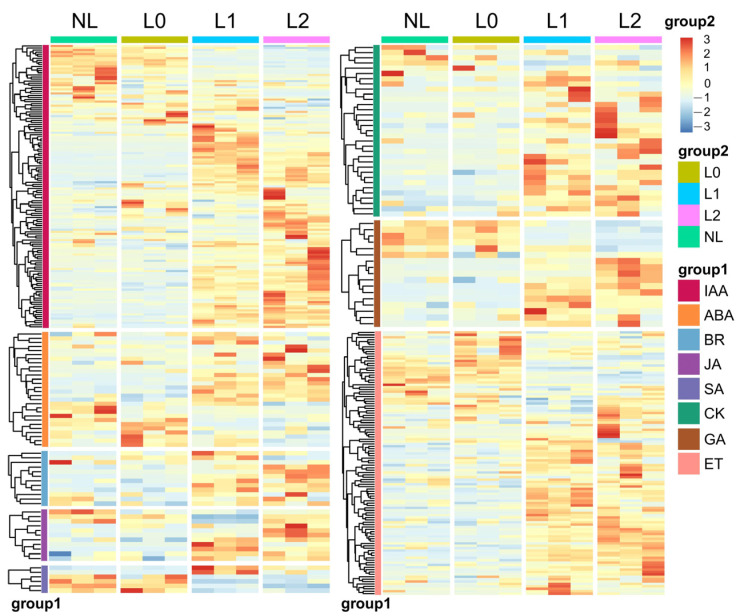
Temporal changes in the expression of phytohormone-related DEGs at the four stages of pitaya growth. Group 1 represent the temporal changes in gene expression related to phytohormone metabolism and signaling at the four stages of IAA, CK, GA, ABA, ET, BR, JA, and SA, and the heatmap shows the relative transcript levels of their metabolism and signaling genes. Group 2 represents different stage samples.

The molecular interactions between the components of the hormone and light signaling pathways have been shown in earlier research [29]. For example, auxin plays a significant role in the elongation of the hypocotyl, stem, and petiole induced by shade. Furthermore, shading causes the plant to allocate more carbon resources to growth, reducing its defenses and making it more vulnerable to pathogen attacks. To determine which phytohormone-related gene expression is involved in altering the growth and development pattern of pitaya under supplemental light, we further screened four stages of the response to pitaya hormone-related DEGs to map heatmaps (Figure 2). The results indicated that the expression patterns of IAA, CK, GA, ABA, ETH, BR, JA, and SA phytohormones exhibited varying degrees of changes following supplemental light (L0) (Figure 2). In particular, the expression of genes related to IAA, CK, JA, and GA was significantly increased by light supplementation, and the hormonal changes were most pronounced from the beginning of the light supplementation phase (L0–L1) to the bud formation phase. This result suggests that supplemental light promote the synthesis and regulation of phytohormones that induce flower formation.

### 2.3. Identification of Differential Phytohormone-Related Metabolites in Pitaya following Supplemental Lighting

**Figure 3:**
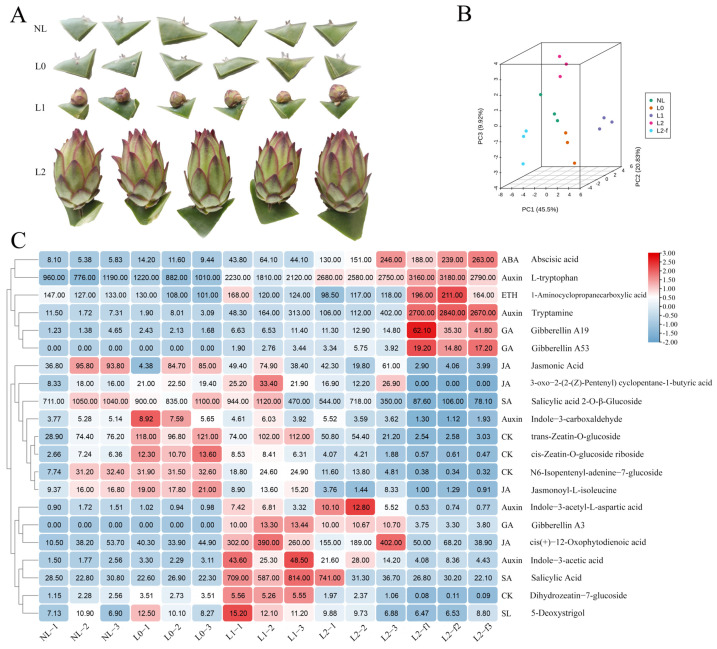
Pitaya hormone-related differential metabolites. (**A**): The picture of metabolome sample. (**B**): Principal component analysis of metabolome samples. Each point represents an independent biological replicate. (**C**): Heat map showing the differential metabolites. In the map, red color indicates increased levels, while blue color indicates decreased levels.

In order to further characterize the phytohormone changes affected by supplemental light conditions in winter, and their role in the development of pitaya from areoles to flower bud, we collected samples from these four periods and used targeted metabolomics to detect changes in the concentration of phytohormones and their contents in pitaya samples under supplemental light (Figure 3A,B). A total of 41 metabolites were identified, with 21 phytohormones showing differentiation (Figure 3C).

The CK (trans-Zeatin-*O*-glucoside, cis-Zeatin-*O*-glucoside riboside) content in pitaya areoles is increased by 1.5–2-fold at the L0 stage relative to the NL stage, suggesting that CK plays an important role in promoting floral formation after light supplementation. Upon entering the L1 stage, GA (Gibberellin19, Gibberellin53, Gibberellin A3) started to increase, indicating the significant role of GA in flower bud formation and development. In addition, at the L2 stage, the contents of ABA and IAA increased significantly, while the contents of JA (3-oxo-2-(2-(Z)-Pentenyl) cyclopentane-1-butyric acid, Jasmonoyl-L-isoleucine, cis(+)-12-Oxophytodienoic acid), SA (Salicylic acid 2-*O*-β-Glucoside), and CK (trans-Zeatin-*O*-glucoside, cis-Zeatin-*O*-glucoside riboside, N6-Benzyladenine-7-glucoside, Dihydrozeatin-7-glucoside) decreased in content (Figure 3C). This result may indicate that pitaya reallocates resources from growth and defense to reproductive development during the L2 stage. 

Through comparing the changes in phytohormones at the L2 and L2f stages, an increase in the content of IAA, GA, ET, and ABA was found. This finding further suggests that these metabolites play a crucial role during flower development. In conclusion, changes in endogenous hormones in pitaya under supplemental light conditions affected the process of flower induction and development. CK and GA may play a decisive role in this process.

### 2.4. Hormone’s Level Correlated with the Gene Expression Levels of Hormone Biosynthesis Transcripts in Pitaya

**Figure 4:**
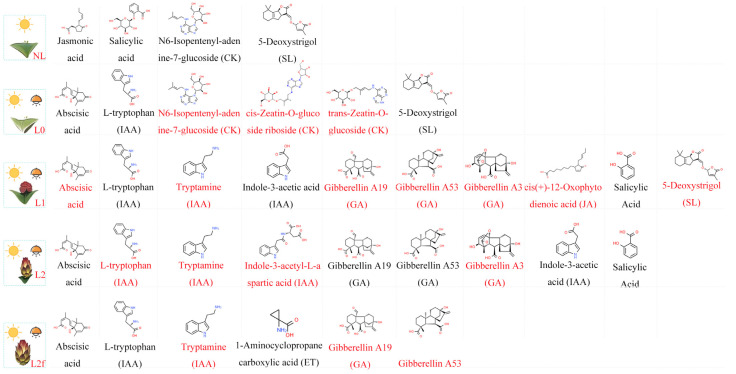
Changes in pitaya hormone expression at different stages under supplemental light conditions. L0, L1, L2, and L2f represent the stages following the application of supplemental light to the pitaya. The red name represents the increase in content at this stage.

**Figure 5:**
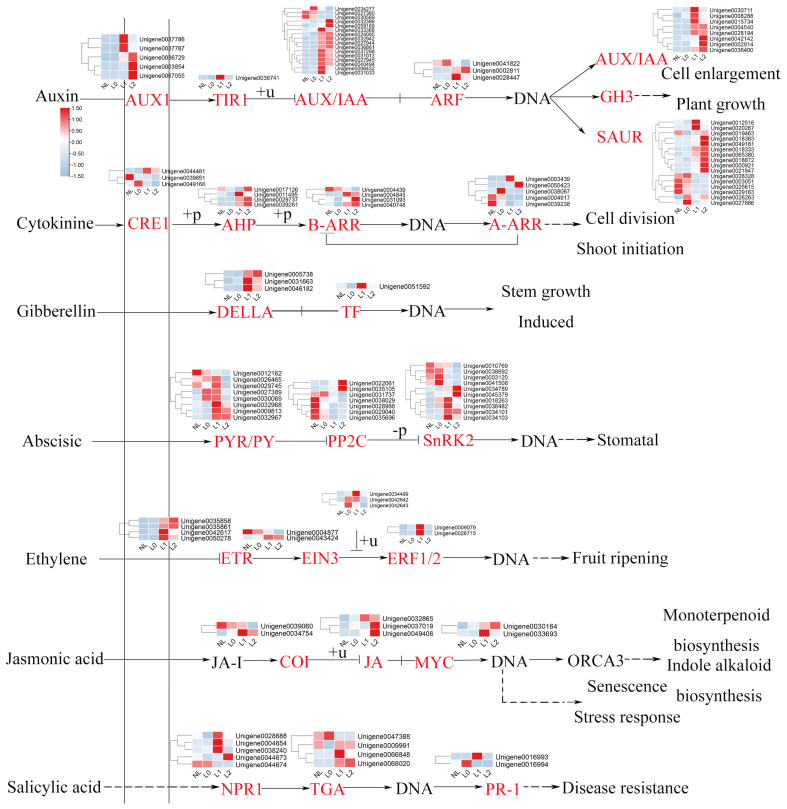
Changes in expression of genes related to the pitaya hormone pathway at different stages under supplemental light conditions. Red font indicates up-regulation of gene expression, and the heatmap shows the expression of relevant genes in the phytohormone signaling pathway that regulates compound synthesis.

**Figure 6:**
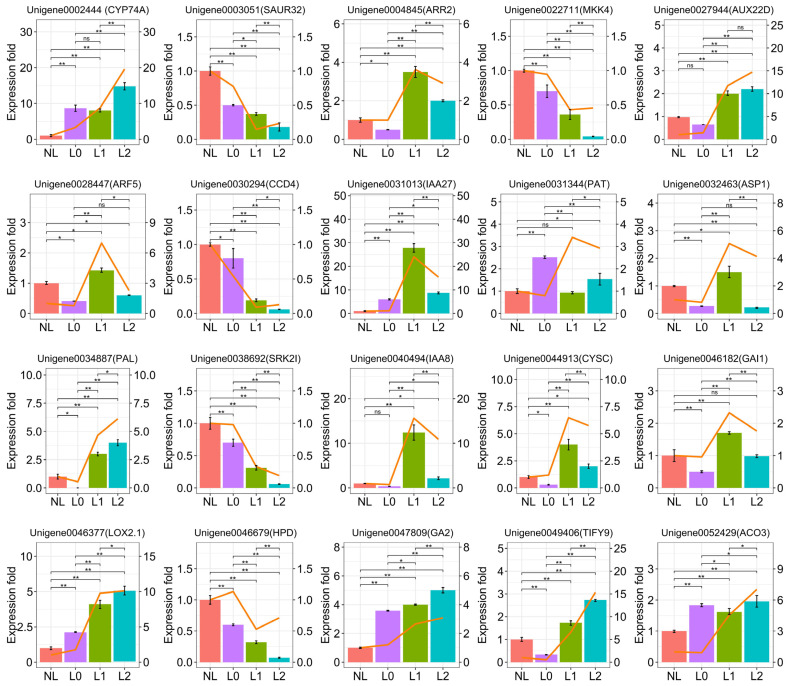
Changes in genes related to plant hormone synthesis pathways under supplemental light conditions. Relative transcript levels of genes involved in plant metabolism and signaling under supplemental light conditions. Bars indicate gene expression values detected by qRT-PCR, while the orange lines represent the gene expression values from transcriptome sequencing. Data are given as means ± SD. Significant differences were determined by *t*-tests using R (version 4.3.3) (ns: no significant difference, * *p* < 0.05, ** *p* < 0.01).

To investigate the changes in the expression of genes involved in the phytohormone biosynthesis pathway during flower formation in pitaya under supplemental light conditions, we focused on identifying DEGs related to phytohormone synthesis. The results showed that these genes were mainly enriched in the phytohormone signaling pathway (ko04075) (Figure 4 and Figure 5). We found that CK content initially increased under supplemental light conditions (Figure 4), and the expression of genes related to the phosphotransfer protein (*AHP*) and *Arabidopsis* Response Regulator (*ARR*) in the CK pathway was up-regulated. The gene associated with *AHP* synthesis (*Unigene0039261*) was up-regulated 2-fold during L0 and 28-fold during L1 (Figure 5). This was verified by qRT-PCR experiments (Figure 6).

At the L1 stage, GA content increases, influencing plant flowering regulation by controlling the levels of DELLA proteins. As shown in Figure 5, DELLA-related genes were up-regulated for expression at the L1 stage. For example, *Unigene0005738* was up-regulated 4-fold, and *Unigene0031663* was up-regulated 8-fold. We also found that the expression of the TF gene *Unigene0051592* (Phytochrome interacting factor3, *PIF3*) was up-regulated. *PIF3* is an essential component of photoreceptor pigment signaling during the initial phases of the change from darkness to light [29].

The most significant increase in IAA content was observed during the L2 and L2f stages (Figure 5), and many genes responsive to IAA were up-regulated for expression during this period. In addition, ABA, SA, JA, and ETH played important roles in pitaya flower development (Figure 4). SA content significantly increased at the L1 stage. SA could regulate the flowering time of the plant by participating in the regulation of the expression of flowering-related genes, such as *CO*, *FLC*, *FT*, and *FLD* in the photoperiod [30]. Therefore, we hypothesized that a large amount of SA was synthesized at the L1 stage concerning the flowering time of pitaya and its adaptation to the external environment. JA-related *MYC2* transcription factors were significantly up-regulated during the L1 period (Figure 5). It has been shown that JA represses the transcription of the *FT* gene through its activated transcription factor *MYC2*/*3*/*4*, thus inhibiting the induction of flowering in plants [20]. Conversely, mutation of *MYC2*/*3*/*4* resulted in an earlier flowering time of the plants. In the ETH signaling pathway, the ETH content was significantly increased at the L2f stage (Figure 3C and Figure 4). Recent studies have indicated that ETH is the primary regulator of plant sex determination in monoecious plants [22]. Therefore, it is crucial to acknowledge that ETH plays a key role in the sex determination of pitaya. 

### 2.5. qRT-PCR Validation

Based on the fact that supplemental light induces the synthesis of phytohormones, leading to flower formation in plants, we verified the expression of genes responsive to these hormones using qRT-PCR. The results revealed the up-regulation of genes responsive to JA-, IAA-, CK-, GA-, and ETH-related hormones. Additionally, we selected several kinases, including MKK4, phenylpropanoid PAL, and transcription factor TIFY9 genes for validation. The results demonstrated that the expression of these genes was consistent with the expression trend observed in the transcriptome data (Figure 6).

## 3. Discussion

Phytohormones are essential intrinsic factors that regulate the development of individual plants throughout their life cycle. In model plants, different phytohormone routes are combined to form a sophisticated network of additive, antagonistic, and synergistic interactions [12]. Pitaya is a long-day plant that requires more than 12 h of sunlight to bloom; otherwise, flowering may be delayed or even absent [27]. However, the geographic location of Hainan, China, in the northern hemisphere does not provide enough winter sunlight for pitaya to thrive. As a result, fruit growers try to compensate for the lack of sunlight in winter by using supplemental lighting. However, this method incurs high costs and results in wastage of electrical resources. Previous studies have shown the molecular mechanism of pitaya flowering induction under supplemental light through transcriptome analysis, and discovered that three hormone-related genes, IAA, JA, and BR, are involved in the signaling pathway of flower formation and floral organ development in plants [27]. However, the current understanding of the relationship between supplemental light and phytohormones, and how phytohormones synergistically or antagonistically regulate flower formation and floral organ development in pitaya, remains incomplete. Therefore, we reanalyzed transcriptomic data from four stages (NL, L0, L1, L2) and combined them with targeted metabolomic analysis to elucidate the changes in supplemental light and phytohormone content, as well as the relationship between them.

A central genetic network in plants controls flowering, and this network is impacted by several internal, external, and seasonal events [31]. In times of adversity, many species accelerate the transition to flowering in order to reproduce before dying. The role of ABA and SA in plant flowering is currently unknown. However, previous evidence suggests that flowering is delayed in the ABA biosynthesis mutant and advanced in the ABA-hypersensitive *pp2c* triple mutant under long-day conditions [32]. By constructing co-expression profiles for analysis, we enriched the up-regulated genes associated with phytohormone-mediated signaling pathways in clusters 41, 39, and 18 (Figure 1B and Figure S1E,F). Further analysis revealed that these genes were functionally associated with responding to stress, response to biotic, and cellular responses to chemical stimulus. Through metabolomic analysis, we observed a significant increase in SA content and a notable rise in ABA content at the L1 stage. This implies that ABA and SA might be involved in the development of floral buds. Additionally, the newly emerged flower buds appeared to be more vulnerable to pests and environmental threats, prompting the plant to release higher levels of SA and ABA in response to pest and disease attacks as well as environmental changes. We found that ETH was mainly present in the flowers and was less abundant in the areoles than in the floral organs. ETH has been found to be a major regulator of plant sex determination, and EHT has been shown to be a key hormone involved in flower sex determination in Cucurbitaceae [22]. As a result, we hypothesize that ethylene may be crucial in pitaya sex determination.

Plant cell division, tissue growth, organ development, nutrient uptake, and responses to biotic and abiotic stressors are all regulated by CK [13]. Our analysis of clusters 28, 29, 21, 20, and 27 revealed an increase in DNA and RNA synthesis and an acceleration of the cell cycle in pitaya (Figure S1A–D,G). Upon analyzing the L0 stage data using metabolomics, we observed an increase in the content of CK. It has been shown that CK accelerates DNA replication in the pre-division phase of cell division and lays the foundation for cell division in the later phase [33]. This suggests that after initial supplementation of light, plants may accelerate the synthesis of CK to promote DNA replication and cell proliferation, facilitating the transition from the trophic stage to the reproductive stage by controlling the changes in plant hormones.

The relationship between the biological clock, temperature, and the growth hormone pathway is complex [34]. By analyzing 48 and 47 clusters, we found that pitaya growth metabolism slowed down while nitrogen metabolism was enhanced. (Figure S1L–M). Additionally, metabolome analysis revealed that the level of IAA increased nearly twofold at the L1 stage compared with L0 and continued to increase. We hypothesized that when pitaya transitioned from the growth stage to the reproductive stage, there was an increased transport of IAA to the stem tip to facilitate the development of floral organs. It was observed that the stem and floral organs exhibited different sensitivities to growth hormone, resulting in slow stem growth and full development of floral organs under the influence of high growth hormone concentration. In addition, we compared the L2 and L2f stages and observed a significant increase in growth hormone content in the floral organs, particularly in the content of tryptophan, which was nearly seven times higher than that in the L2 stage (Figure 3C). This indicates that the synthesis of growth hormone in pitaya mainly depends on the tryptophan pathway.

Additionally, GA is a crucial hormone involved in seed germination, and flower induction and development [12]. Research has demonstrated that GA actively participates in the floral transition of *Arabidopsis thaliana* [34]. In this study, GA was found to be increased in L1 content and persisted in the subsequent stages of floral organ development, indicating that GA plays an essential role in floral organ development (Figure 4). By analyzing the phytohormone pathway, we discovered that *DELLAs* were up-regulated for expression at L1. DELLAs can delay flowering by inhibiting the TF CONSTANS, which stimulates flowering [16]. DELLA proteins are central nodes in GA signaling, interacting with SPL3/4 to induce AP1 and indirectly induce LFY, which collectively promote flower development. This suggests that DELLA proteins play an important role in inducing flowering development.

In addition, there is antagonism between GA and CK that affects many developmental processes [12]. In *Arabidopsis*, GA inhibits the response to CK through SPY. DELLAs are crucial repressors of GA signaling. In turn, GA signaling partially inactivates DELLAs by triggering their proteasomal degradation [35,36]. DELLAs interact with ABI3 and ABI5, and together these protein complexes stimulate the transcription of *SOMNUS* that is crucial for dormancy promotion in tissues [36]. This activation leads to the expression of ABA biosynthesis genes and the suppression of GA biosynthesis genes. By comparing GA and CK in L2 and L2f stages, we found that GA and CK also play antagonistic roles during pitaya development Figure 3C and Figure 4). It is worth mentioning that naturally occurring GA4 is the most active GA during flower induction. Additionally, the contents of GA19 and GA53 are higher than those of GA3 in pitaya, suggesting that GAs have different roles in promoting flower development in pitaya (Figure 3C).

**Figure 7:**
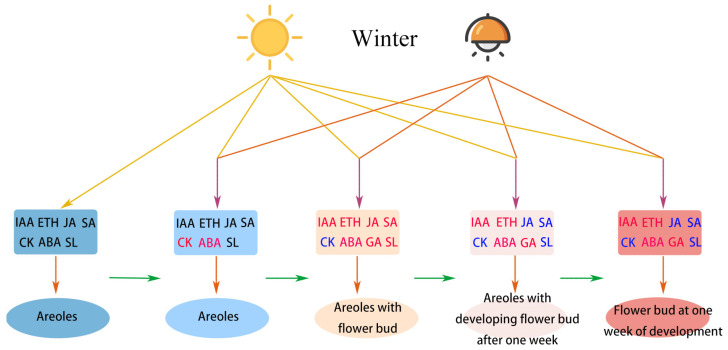
Schematic modeling of light–hormone interactions during the development of pitaya with winter supplementation. Solid lines indicate the direct injection of a process. Hormones in black font indicate no change in content, red font indicates an increase in content, and blue font indicates a decrease in content.

In conclusion, pitaya regulates the flowering and developmental processes of plants in a simultaneous and synergistic manner through the interaction of light and hormones during winter supplementation (Figure 7). At the L0 stage, CK increased, accelerating pitaya’s DNA and RNA synthesis to speed up the cell cycle. With the accumulation of organic matter, pitaya transitioned from the development stage to the reproductive stage. At the L1 stage, GA and IAA were increased to promote the formation and rapid development of flower buds. Additionally, pitaya also promoted the synthesis of SA and ABA to protect pitaya flower buds from pests, diseases, and environmental impacts. During the L2 stage, GA and IAA continued to be synthesized to promote the development of pitaya floral organs. During the L2f stage, pitaya required a higher concentration of IAA to promote flower growth. Furthermore, there was an increase in the amounts of GA, ET, and ABA, suggesting that GA is important for flower development and growth, and that ABA and ETH are important for pitaya’s response to biotic and abiotic stressors. Moreover, ETH is particularly important in the sex determination process in pitaya. 

## 4. Materials and Methods

### 4.1. Plant Material

The one-year-old “Jindu No. 1” pitaya (*Hylocereus polyrhizus*), a widely cultivated variety in China, was chosen as the plant material for this study. This variety was selected through hybridization of the progeny. The plants were grown in the orchard of Enhong Agricultural Science and Technology Co., located in Banqiao Town, Dongfang City, Hainan Province, China. The samples were collected in December under different conditions: from pitaya areoles that were treated with supplemental light but did not show flower buds (L0); after one month of supplemental lighting, the areoles on the pitaya were collected (L1); the pitaya areoles after one week of flower bud development were collected under supplemental light (L2); and the L2′s flower buds were collected under supplemental light (L2f). As a control, pitaya areoles that did not receive additional light (NL) were employed. We mixed at least ten samples into one sample. Each sample was then be subjected to three biological replicates.

### 4.2. Pitaya Supplemental Light Conditions

**Figure 8:**
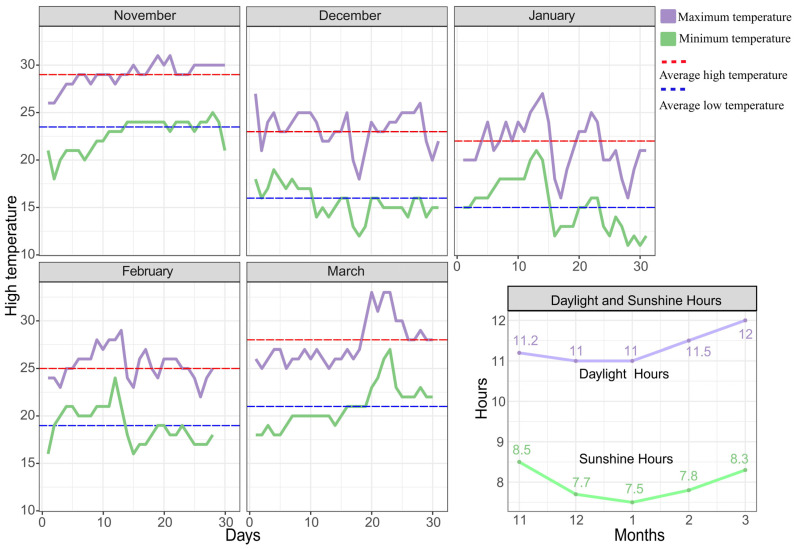
Statistics on maximum temperature, minimum temperature, daytime hours, and sunshine hours from November to March in Dongfang City, Hainan Province.

In field cultivation, the period from November to March is typically selected for providing pitaya with supplemental light. We recorded the temperature and sunshine duration from November to March 2022 in Dongfang City, Hainan Province, and the results are shown in Figure 8. In this experiment, we used the same supplemental light conditions as in Xiong Rui’s study [27]. We selected a 15W LED hybrid bulb as the light source, set the spectral ratio of red light to blue light to 7:1, and regulated the color temperature within the range of 2900–3300 K. In addition, pitaya in the experimental group received supplemental light from 6:30 p.m. until 11:30 p.m.; meanwhile, plants in the control group were not treated with supplemental light.

### 4.3. Transcriptomic Data and Co-Expression Trend Analysis

Transcriptomic data were obtained from the publicly available NCBI database (GSE125083). First, the number of mapped reads and the transcript length in each of the four samples were normalized to make sure the number of fragments appropriately reflects the transcript expression level. To assess transcript or gene expression levels, StringTie’s maximum flow technique was used to normalize the FPKM (fragments per kilobase of transcript per million fragments mapped). The DESeq2 (version 1.42.1) program was used to carry out the difference analysis [37]. Two of the screening criteria were FDR < 0.01 and Fold Change ≥ 2. The fold change indicates the expression ratio between two samples. By modifying the *p*-value to take the significance of differences into account, the False Discovery Rate (FDR) was computed, thereby highlighting the significance of differences. STEM software (https://www.stem.com/ (accessed on 12 December 2023) was used to assess differentially expressed genes (DEGs) having an absolute logFC value of ≥2 [38]. The parameters were as follows: 20 output profiles were the maximum, and profiles that were similar were combined. Using the TBtools software (version 2.085) program, the genes were examined for GO enrichment and then visualized and heatmapped [39].

### 4.4. Quantitative Analysis of Plant Hormones

Five phases of samples were used to measure ABA, SA, IAA, JA, GA, SL, CK, and ETH. The samples were first pulverized into a powder. Following the weighing of 50 mg of the ground sample, the appropriate quantity of the internal standard was applied. One milliliter of methanol, water, and formic acid (15:4:1, *v*:*v*:*v*) was used to extract the combination. After being diluted and concentrated in 100 μL of 80% methanol/water solution, the extracts were run through a 0.22 μm filter membrane. The final extract was put in the injection bottle for LC-MS/MS analysis after it had been concentrated and redissolved in 100 μL of an 80% methanol/water solution. The following were the primary liquid-phase conditions: Waters ACQUITY UPLC HSS T3 C18 column (1.8 µm, 100 mm × 2.1 mm i.d.) is the first chromatographic column. The gradient elution program consists of two mobile phases: phase A, ultrapure water with 0.04% acetic acid added, and phase B, acetonitrile with 0.04% acetic acid added. The flow rate is 0.35 mL/min, the column temperature is 40 °C, and the injection volume is 2 μL. Timings and ratios were set as 8.0 min at 5:95 (*v*/*v*), 9.0 min at 5:95 (*v*/*v*), 9.1 min at 95:5 (*v*/*v*), and 12.0 min at 95:5 (*v*/*v*). The primary parameters for mass spectrometry were as follows: a temperature of 550 °C for electrospray ionization (ESI), a voltage of 5500 V for positive ion mode, a voltage of −4500 V for negative ion mode, and a pressure of 35 psi for the curtain gas. Every ion pair in the Q-Trap 6500+ was tuned using the declustering potential (*v*/*v*) optimization. Using optimal collision energy (CE) and declustering potential (DP), every ion pair in the Q-Trap 6500+ was scanned. In accordance with accepted practices, a Metware Database (MWDB) was created for the qualitative examination of mass spectrometry data. Triple quadrupole mass spectrometry’s Multiple Reaction Monitoring (MRM) mode was used for the quantification.

### 4.5. Differential Metabolite Screening and Annotation

The metabolites with fold changes of at least two and at least half of five were chosen as the final difference metabolites. The difference multiplicity value (fold change) was computed, and the *p*-value was obtained using the *t*-test or Wilcoxon rank sum test technique. The KEGG database was used to annotate and display differential metabolites [40].

### 4.6. Quantitative Real-Time qRT-PCR

As previously mentioned, quantitative real-time PCR (qRT-PCR) was carried out [25]. Utilizing SYBR^®^Premix Ex Taq (Vazyme, Nanjing, China), fluorescence qRT-qPCR was carried out. The reaction mixture included 1 µL of cDNA (10×), 0.5 µL of primers upstream and downstream, and 10 µL of buffer that was diluted with water to make 20 µL. Table S1 contains a list of the target gene primers that were created with Primer Premier 5. For every sample, there were three biological and three technical replicates. For data analysis, the 2^−ΔΔCt^ method was employed. The internal reference genes were pitaya ubiquitin genes.

## 5. Conclusions

Supplemental light at night in winter can interact with endogenous hormone signaling in pitaya, especially CK, which regulates bud formation, and GA, which promotes bud development. These results contribute to a better understanding of the mechanisms of phytohormone interactions during the induction of flowering in pitaya under supplemental winter light. It provides theoretical guidance for the rational use of phytohormones in production. Meanwhile, these studies will help us examine the changes and applications of hormones in various plants during flower formation.

## Data Availability

Data is contained within the article and Supplementary Materials.

## Supplementary Materials

The following supporting information can be downloaded at: https://www.mdpi.com/article/10.3390/ijms25094794/s1.
