# Supplementary material for: Light Supplementation in Pitaya Orchards Induces Pitaya Flowering in Winter by Promoting Phytohormone Biosynthesis
Source: Int J Mol Sci. 2024 Apr 27;25(9):4794. doi: 10.3390/ijms25094794 (PMC11083671; doi:10.3390/ijms25094794)
Supplement: Supplementary file 1 [file ijms-25-04794-s001.zip › Figure S1.pdf]

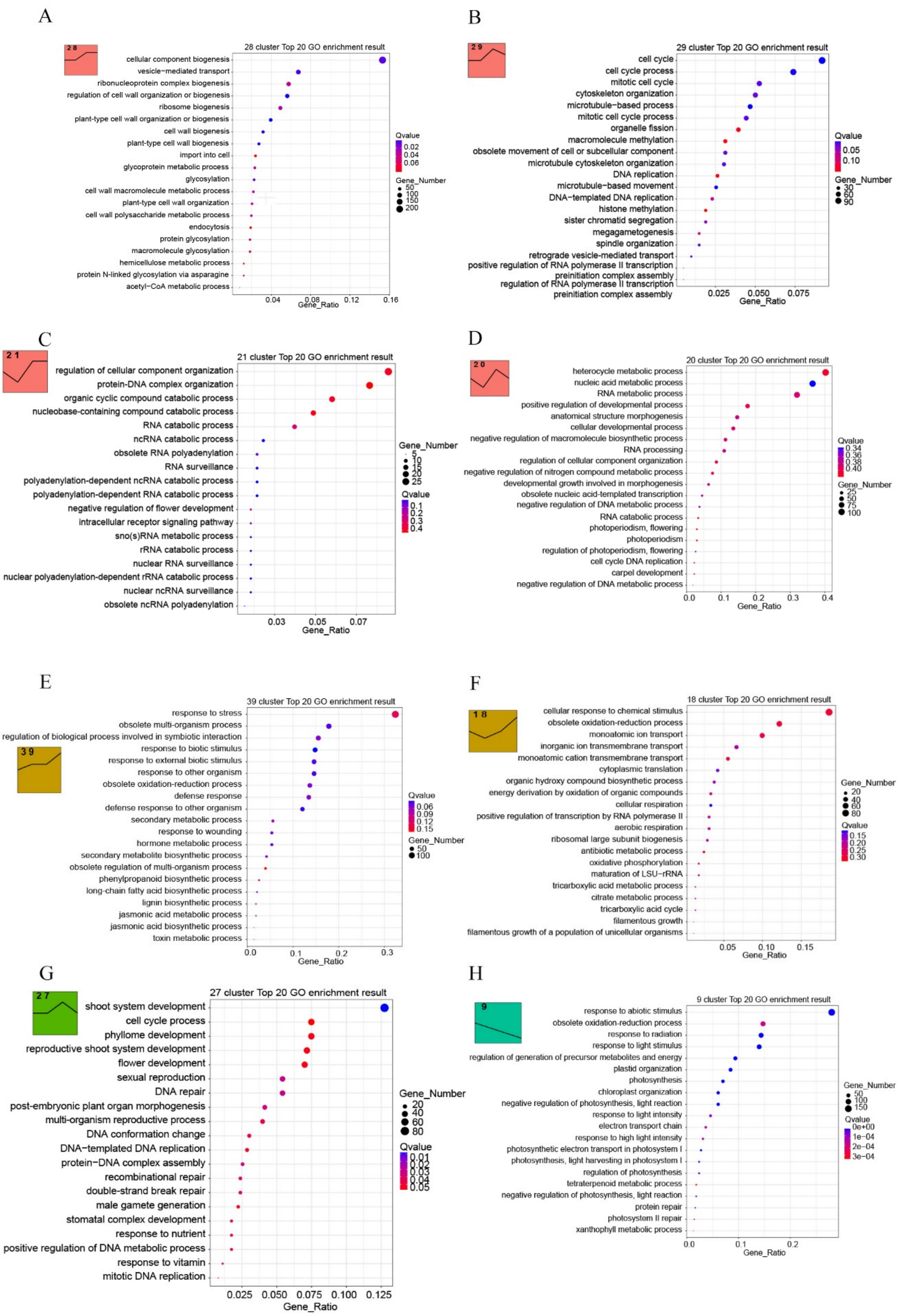
A
28
28 cluster Top 20 GO enrichment result
cellular component biogenesis
vesicle−mediated transport
ribonucleoprotein complex biogenesis
regulation of cell wall organization or biogenesis
ribosome biogenesis
plant-type cell wall organization or biogenesis
cell wall biogenesis
plant-type cell wall biogenesis
import into cell
glycoprotein metabolic process
glycosylation
cell wall macromolecule metabolic process
plant-type cell wall organization
cell wall polysaccharide metabolic process
endocytosis
protein glycosylation
macromolecule glycosylation
hemicellulose metabolic process
protein N-linked glycosylation via asparagine
acetyl−CoA metabolic process
0.04
0.08
0.12
0.16
Gene_Ratio
Qvalue
0.02
0.04
0.06
Gene_Number
50
100
150
200
B
29
29 cluster Top 20 GO enrichment result
cell cycle
cell cycle process
mitotic cell cycle
cytoskeleton organization
microtubule−based process
mitotic cell cycle process
organelle fission
macromolecule methylation
obsolete movement of cell or subcellular component
microtubule cytoskeleton organization
DNA replication
microtubule−based movement
DNA−templated DNA replication
histone methylation
sister chromatid segregation
megagametogenesis
spindle organization
retrograde vesicle-mediated transport
positive regulation of RNA polymerase II transcription preinitiation complex assembly
regulation of RNA polymerase II transcription preinitiation complex assembly
0.025
0.050
0.075
Gene_Ratio
Qvalue
0.05
0.10
Gene_Number
30
60
90
C
21
21 cluster Top 20 GO enrichment result
regulation of cellular component organization
protein-DNA complex organization
organic cyclic compound catabolic process
nucleobase-containing compound catabolic process
RNA catabolic process
ncRNA catabolic process
obsolete RNA polyadenylation
RNA surveillance
polyadenylation-dependent ncRNA catabolic process
polyadenylation-dependent RNA catabolic process
negative regulation of flower development
intracellular receptor signaling pathway
sno(s)RNA metabolic process
rRNA catabolic process
nuclear RNA surveillance
nuclear polyadenylation-dependent rRNA catabolic process
nuclear ncRNA surveillance
obsolete ncRNA polyadenylation
0.03
0.05
0.07
Gene_Ratio
Gene_Number
5
10
15
20
25
Qvalue
0.1
0.2
0.3
0.4
D
20
20 cluster Top 20 GO enrichment result
heterocycle metabolic process
nucleic acid metabolic process
RNA metabolic process
positive regulation of developmental process
anatomical structure morphogenesis
cellular developmental process
negative regulation of macromolecule biosynthetic process
RNA processing
regulation of cellular component organization
negative regulation of nitrogen compound metabolic process
developmental growth involved in morphogenesis
obsolete nucleic acid-templated transcription
negative regulation of DNA metabolic process
RNA catabolic process
photoperiodism, flowering
photoperiodism
regulation of photoperiodism, flowering
cell cycle DNA replication
carpel development
negative regulation of DNA metabolic process
0.0
0.1
0.2
0.3
0.4
Gene_Ratio
Qvalue
0.34
0.36
0.38
0.40
Gene_Number
25
50
75
100
E
39
39 cluster Top 20 GO enrichment result
response to stress
obsolete multi-organism process
regulation of biological process involved in symbiotic interaction
response to biotic stimulus
response to external biotic stimulus
response to other organism
obsolete oxidation-reduction process
defense response
defense response to other organism
secondary metabolic process
response to wounding
hormone metabolic process
secondary metabolite biosynthetic process
obsolete regulation of multi-organism process
phenylpropanoid biosynthetic process
long-chain fatty acid biosynthetic process
lignin biosynthetic process
jasmonic acid metabolic process
jasmonic acid biosynthetic process
toxin metabolic process
0.0
0.1
0.2
0.3
Gene_Ratio
Qvalue
0.06
0.09
0.12
0.15
Gene_Number
50
100
F
18
18 cluster Top 20 GO enrichment result
cellular response to chemical stimulus
obsolete oxidation-reduction process
monoatomic ion transport
inorganic ion transmembrane transport
monoatomic cation transmembrane transport
cytoplasmic translation
organic hydroxy compound biosynthetic process
energy derivation by oxidation of organic compounds
cellular respiration
positive regulation of transcription by RNA polymerase II
aerobic respiration
ribosomal large subunit biogenesis
antibiotic metabolic process
oxidative phosphorylation
maturation of LSU−rRNA
tricarboxylic acid metabolic process
citrate metabolic process
tricarboxylic acid cycle
filamentous growth
filamentous growth of a population of unicellular organisms
0.05
0.10
0.15
Gene_Ratio
Gene_Number
20
40
60
80
Qvalue
0.15
0.20
0.25
0.30
G
27
27 cluster Top 20 GO enrichment result
shoot system development
cell cycle process
phyllome development
reproductive shoot system development
flower development
sexual reproduction
DNA repair
post-embryonic plant organ morphogenesis
multi-organism reproductive process
DNA conformation change
DNA−templated DNA replication
protein−DNA complex assembly
recombinational repair
double-strand break repair
male gamete generation
stomatal complex development
response to nutrient
positive regulation of DNA metabolic process
response to vitamin
mitotic DNA replication
0.025
0.050
0.075
0.100
0.125
Gene_Ratio
Gene_Number
20
40
60
80
Qvalue
0.01
0.02
0.03
0.04
0.05
H
9
9 cluster Top 20 GO enrichment result
response to abiotic stimulus
obsolete oxidation-reduction process
response to radiation
response to light stimulus
regulation of generation of precursor metabolites and energy
plastid organization
photosynthesis
chloroplast organization
negative regulation of photosynthesis, light reaction
response to light intensity
electron transport chain
response to high light intensity
photosynthetic electron transport in photosystem I
photosynthesis, light harvesting in photosystem I
regulation of photosynthesis
tetraterpenoid metabolic process
negative regulation of photosynthesis, light reaction
protein repair
photosystem II repair
xanthophyll metabolic process
0.0
0.1
0.2
Gene_Ratio
Gene_Number
50
100
150
Qvalue
0e+00
1e−04
2e−04
3e−04

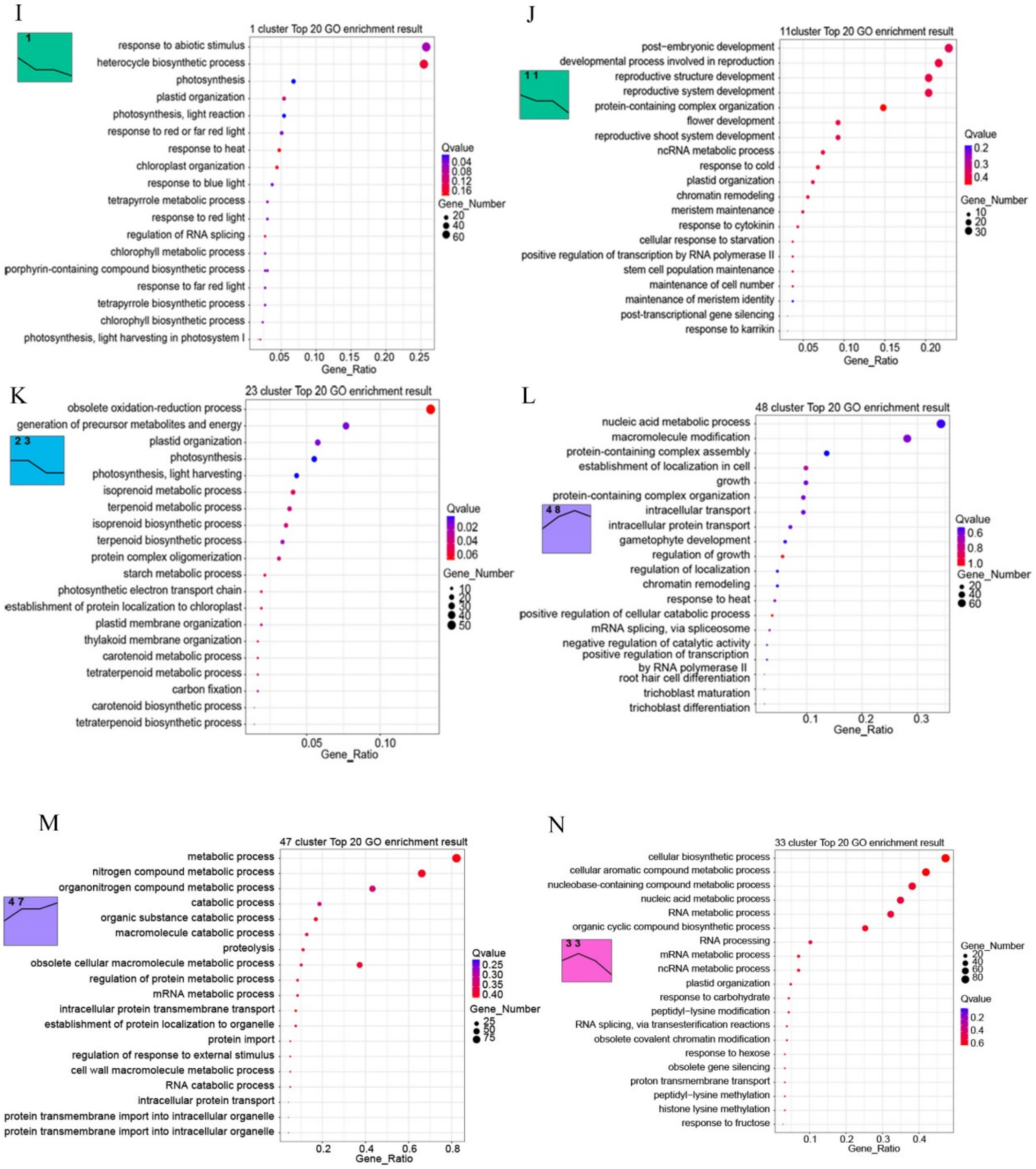

Figure S1 A-D: GO enrichment analysis of clusters 28, 29, 21, and 20; E-H: GO enrichment analysis of clusters 39, 18, 27, and 9; I-L: GO enrichment analysis of clusters 1, 11, 23, and 48; M-N: GO enrichment analysis of clusters 47 and 33.
